# Identification of exosome miRNAs regulated by SPHK2 in human glioma cells

**DOI:** 10.3389/fonc.2025.1662312

**Published:** 2025-12-16

**Authors:** Jing Liu, Huafu Zhao, Xia Liu, Xiaoyun Guan, Qiyu Guo, Yanwen Xu, Jinhua Qiu

**Affiliations:** 1 Departments of Pathology and Translational Medicine Institute, Shenzhen Second People’s Hospital, The First Affiliated Hospital of Shenzhen University Health Science Center, Shenzhen, Guangdong, China; 2 Department of Neurology, Huizhou First Hospital, Huizhou, Guangdong, China

**Keywords:** glioma, macrophage, sphingosine kinase 2, exosome, miRNA

## Abstract

**Background:**

Glioma is the most common primary brain tumor, and the WHO 4 glioma, glioblastoma (GB), is a malignant tumor with high invasiveness and mortality. Tumor-associated macrophages (TAMs), as the main immune cells in glioma, play an important role in the growth, invasiveness, immune escape, and drug resistance. The M2 phenotype of macrophages, but not the M1 phenotype, promotes glioma development. Recent studies have shown that sphingosine kinase 2 (SPHK2) is positively associated with TAM infiltration and glioma proliferation. SPHK2-deficient tumors showed impaired growth and failed to polarize macrophages toward an M2 phenotype.

**Objective:**

Our aim was to reveal whether SPHK2 affects exosome microRNA (miRNA) release from glioma cells and which miRNAs regulated by SPHK2 could mediate the polarization of macrophages around glioma cells.

**Methods:**

SPHK2 knockdown of the human glioma cell line U373 was performed using short hairpin RNA (shRNA) lentiviruses. Exosome miRNAs were isolated and evaluated using RNA sequencing (RNA-seq). Gene Ontology (GO) analysis was carried out to determine the function of exosome miRNAs targeting genes. Kyoto Encyclopedia of Genes and Genomes (KEGG) analysis was used to view the pathways of these genes.

**Results:**

We successfully isolated exosomes from the U373-HK and U373-SH glioma cell lines. We found 12 exosome miRNAs differentially expressed between U373-HK and U373-SH cells. The heatmap showed two upregulated and 10 downregulated miRNAs in glioma cells with SPHK2 knockdown. There were 12 miRNAs targeting genes assigned to 118 GO terms, including 53 biological processes, 30 cellular components, and 35 molecular function terms. KEGG mapping further clustered several signaling pathways, such as “Wnt signaling,” “p53 signaling,” “proteoglycans in cancer,” and “cell cycle.” “Wnt signaling” was identified as the most significantly enriched pathway in the KEGG analysis. A total of 17 genes were enriched in this pathway.

**Conclusions:**

The present study elucidates that SPHK2 promoted the release of 10 exosome miRNAs, but inhibited the release of two miRNAs. The KEGG data indicated that these miRNAs targeted several important genes of Wnt signaling. This study provides not only a genomic resource for further studies but also novel insights into uncovering the molecular mechanism of SPHK2 regulating M2 TAM polarization.

## Introduction

Glioma is the most common primary brain tumor, and the WHO 4 grade glioma, glioblastoma (GB), is a malignant tumor with high invasiveness and mortality (1). GB has characteristics of special location, infiltrative growth, difficult operation, and poor efficiency of chemoradiotherapy, with the median survival of the majority of patients being less than 1 year after administration of multimodal therapies (2, 3). For many years, studies on the characteristics and the molecular mechanisms of the occurrence, progression, and prognosis of malignant glioma have progressed, but there is no breakthrough in the treatment of glioma. In recent years, studies have increasingly shown that tumor resistance to treatment is not necessarily an internal problem, and the interaction between glioma and the tumor microenvironment also makes the disease resistant to drugs (4–6). It is known that, in the course of tumor development, a large number of immune cells congregate in the tumor, the majority of which are tumor-associated macrophages (TAMs). Eventually, the majority of these are activated to anti-inflammatory macrophages (M2 macrophages), but not pro-inflammatory macrophages (M1 macrophages), for the construction of a supportive microenvironment to promote tumor growth (4–6).

Recent studies have shown that sphingosine kinase 2 (SPHK2) plays a role in the phenotypes and function alterations of TAMs (7, 8). TAMs from SPHK2-deficient tumors display a pronounced antitumor phenotype, showing an increased expression of pro-inflammatory markers/mediators such as nitric oxide (NO), tumor necrosis factor alpha (TNF-α), interleukin 12 (IL-12), and major histocompatibility complex II (MHCII) and a low expression of the anti-inflammatory IL-10 and CD206 (9). Our previous study found that the expression level of SPHK2, the macrophage marker CD68, and the M2-type TAM (anti-inflammatory tumor-promoting) markers in glioma tissues were higher than those in the control brain tissue (10). We also verified that miR-137 is a diagnostic tumor-suppressive microRNA (miRNA) that targets SPHK2 to promote M1-type TAM polarization, which successfully established glioma cell lines with SPHK2 knockdown using the lentiviral infection method (11). These studies indicate that SPHK2 could regulate the phenotype polarization of TAMs; however, the mechanism has not been elucidated.

In recent years, research on the exosome in tumors has been increasing, and more and more studies have shown that the exosome derived from tumor cells can promote tumor growth (12). With the exosome as a carrier of “cancer seed” dissemination, its secretion can be used as a clinical diagnostic marker and plays a crucial role in the maturation and secretion of intracellular composition (12). The exosome has an effect on the intercellular material and on information transduction, which can affect tumor growth through antitumor immunity, inhibition of immune function, and mediating tumor immune escape (13). Recent research has shown that SPHK2 mediating the S1P signaling pathway activates the maturation of secretion, molecular loading, and secretion, which is important in many biological functions of glioma (14), suggesting that SPHK2 may affect macrophage polarization through exosome release and function.

Exosomes contain DNAs, proteins, messenger RNAs (mRNA), miRNAs, and many other substances. Among these, miRNAs are the most widely studied substances. Numerous studies have shown that the development of a tumor is often accompanied by the abnormal secretory events of the exosome and exosome miRNAs (15). It is possible to identify an abnormal event in a certain tumor, and it is possible to obtain reliable diagnostic markers and therapeutic targets (16).

At present, the research on the role of the exosome or exosome miRNA secretion in malignant glioma and its effect on the microenvironment of tumors is still in its infancy.

Based on the literature and our previous research, in this study, we demonstrate whether and which exosome miRNAs SPHK2 regulates in glioma cells. This article will display the exosome miRNAs possibly associated with the mechanism of TAM polarization mediated by SPHK2 and will aid in exploring the interaction between glioma and the microenvironment, the formation mechanism of the microenvironment, and useful targets and strategies for glioma treatment.

## Materials and methods

### Cell lines and cell culture

The human glioma cell lines U251 and U373 and normal human astrocyte (HA) were purchased from China Academia Sinica Cell Repository (Shanghai, China). The cells were cultured in Dulbecco’s modified Eagle’s medium (DMEM) (Gibco, Carlsbad, CA, USA) with 10% fetal bovine serum (FBS; Gibco) at 37°C with 5% CO_2_.

### Exosome isolation from cells

Approximately 2 × 10^6^ of the glioma cell lines U373 and U251 were seeded in 10 ml DMEM supplemented with 10% no-exosome FBS. After 48 h, the exosomes were isolated from the cell medium using the RiboTM Exosome Isolation Reagent (Ribobio, Shanghai, China).

### Nanoparticle tracking analysis

Nanoparticle tracking analysis (NTA) measurements were performed using NTA with ZetaView PMX110 (Particle Metrix, Meerbusch, Germany) and the corresponding software ZetaView. The samples were processed in duplicate and diluted using 1× phosphate-buffered saline (PBS) buffer before analysis. The detailed operation procedure was carried out according to the instruction manual.

### Electron microscopy

The exosomes were isolated from the cell medium using the RiboTM Exosome Isolation Reagent (Ribobio, Shanghai, China). The exosome suspension was fixed with paraformaldehyde. Thereafter, 5 μl of the suspended droplets was fixed with a copper net and placed at room temperature for 20 min. Tweezers were used to hold the copper net and for rinsing in the PBS drops. Subsequently, 50 μl of 1% glutaraldehyde was added to the copper net and fixed for 5 min. The copper net was then rinsed with deionized water, 50 μl of uranium acetate was added, and fixed for 5 min. A 50-μl mixture of uranyl acetate and methylcellulose was added on the copper net and fixed on ice for 10 min. A filter paper was used to remove the floating liquid and dried at room temperature for 5–10 min. Images were recorded on a transmission electron microscope (TEM) (JEM-1200EX, Jeol, Tokyo, Japan).

### Lentivirus and stable sub-cell line construction

Empty vector lentiviruses (vector) and lentiviruses expressing SPHK2 short hairpin RNAs (shRNAs) (SPHK2-SH1, SPHK2-SH2, and SPHK2-SH3) with puromycin resistance were constructed and packed by Hanbio (Shanghai, China). The titers of the viral solutions were 1.0–1.2 × 10^9^/ml. U373 and U251 cells were infected with 50 μl of the lentivirus solution per 6-cm dish. At 48 h after infection, the cells were treated with 8 μg/ml puromycin for 14 days for the selection of stable sub-cell lines (vector, SPHK2-SH1, SPHK2-SH2, and SPHK2-SH3).

### Exosome RNA isolation, cDNA library construction, and RNA sequencing

Total RNA was used for the construction of a small RNA (sRNA) library. Briefly, the 3′ and 5′ RNA adapters were ligated to the RNA molecules, followed by reverse transcription to synthesize the first-strand complementary DNA (cDNA). The resulting cDNA was then amplified by PCR. The final cDNA libraries, enriched with inserts of approximately 18–40 nucleotides (nt), were selected by size using gel electrophoresis and quantified. Sequencing was performed on an Illumina HiSeq 2500 platform to generate 50-nt single-end raw reads. For data processing, the raw reads were initially processed to remove adapter sequences and low-quality reads (e.g., those with ambiguous bases or low-quality scores). The resulting clean reads were used for subsequent analysis. The length distribution of these clean reads and the common sequences shared between samples were analyzed. These clean reads were then aligned and annotated against reference databases (e.g., miRBase and Rfam) to identify known RNA species (such as miRNAs and piRNAs, among others) and to quantify their expression. Finally, the unannotated reads were subjected to novel miRNA prediction using tools such as miRDeep2.

### Gene Ontology and Kyoto Encyclopedia of Genes and Genomes pathway mapping analyses

The target genes of the differentially expressed miRNAs were predicted using a combination of two databases, TargetScan and miRDB, to enhance prediction reliability. The genes identified by both algorithms were considered high-confidence targets for subsequent functional analysis. Functional annotation of these target genes was performed using the DAVID Bioinformatics Resource. Gene Ontology (GO) enrichment analysis was conducted to identify the significantly overrepresented terms in the biological process and cellular component domains, with a significance threshold of *p* < 0.05. In addition, Kyoto Encyclopedia of Genes and Genomes (KEGG) pathway analysis was employed to delineate the key biochemical and signal transduction pathways the target genes are involved in using a more stringent threshold of *p* < 0.01.

### Statistical analyses

Normality of the distributions was estimated using the Kolmogorov–Smirnov test. Differences among/between sample groups were analyzed using one-way ANOVA or Student’s *t*-test with SPSS 18.0 software. Data are presented as the mean ± standard deviation (SD). Statistical significance was assigned at *p < 0.05, **p < 0.01, or ***p < 0.001. All the experiments of the cell lines were performed at least three times with triplicate samples.

## Results

### Characterization of isolated exosomes

**Figure 1:**
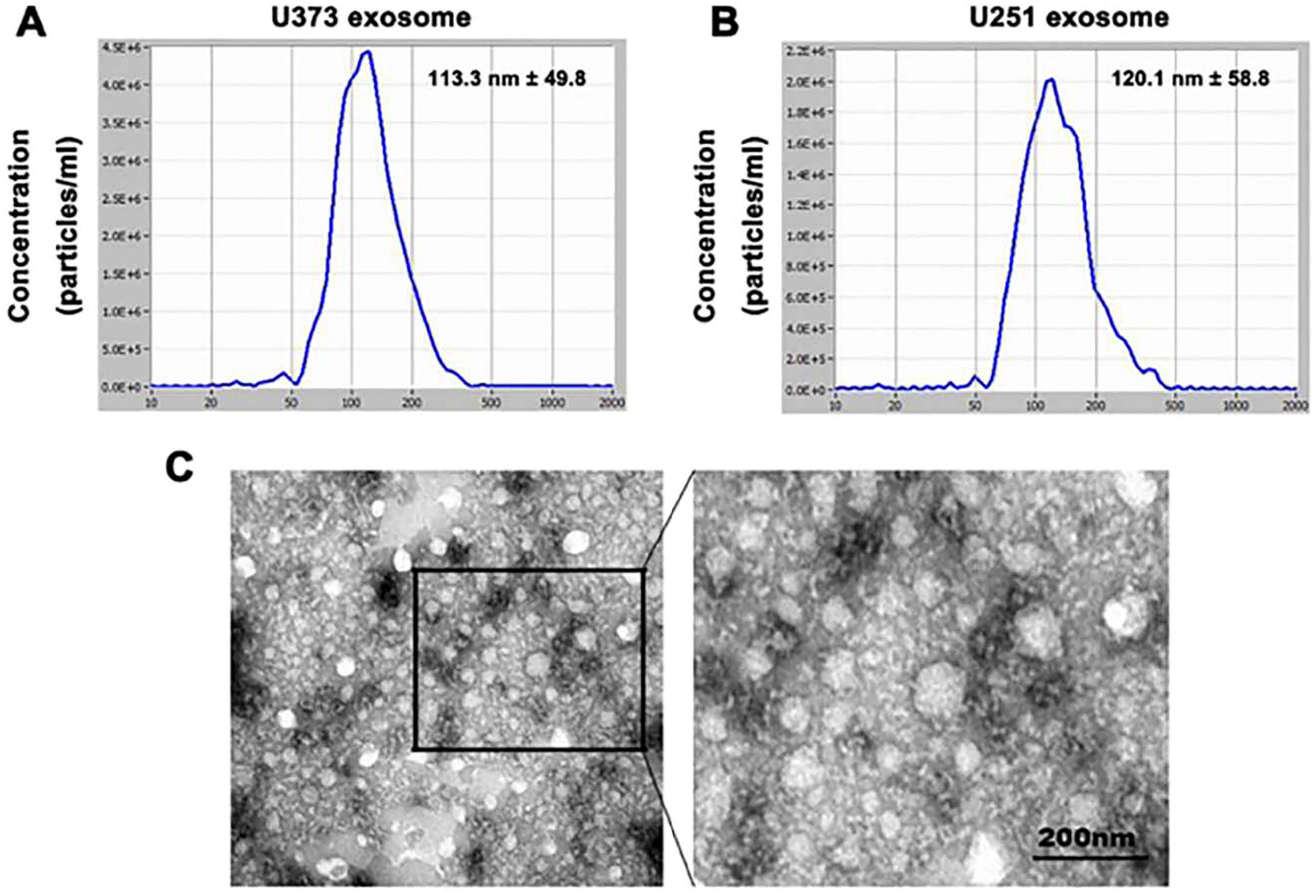
Characterization of the isolated exosomes. **(A, B)** Exosomes from the medium of the glioma cell lines U373 and U251 examined using nanoparticle tracking analysis. **(C)** Exosomes isolated from the U251 supernatant visualized by TEM.

Exosomes were successfully isolated from the cell medium of HA, U373, and U251. These were examined using NTA (**Figures 1A, B**). The results showed that the exosomes from the U373 cell medium had a mean particle diameter of 113.3 ± 49.8 nm, while that of the exosomes from the U251 cell exosome sample was 120.1 ± 58.8 nm. The exosomes isolated from the U251 supernatant were visualized by TEM as nanovesicles ranging in size from 50 to 150 nm (**Figure 1C**).

### Identification of differentially expressed exosome miRNAs between U373-HK and U373-SH cells

**Figure 2:**
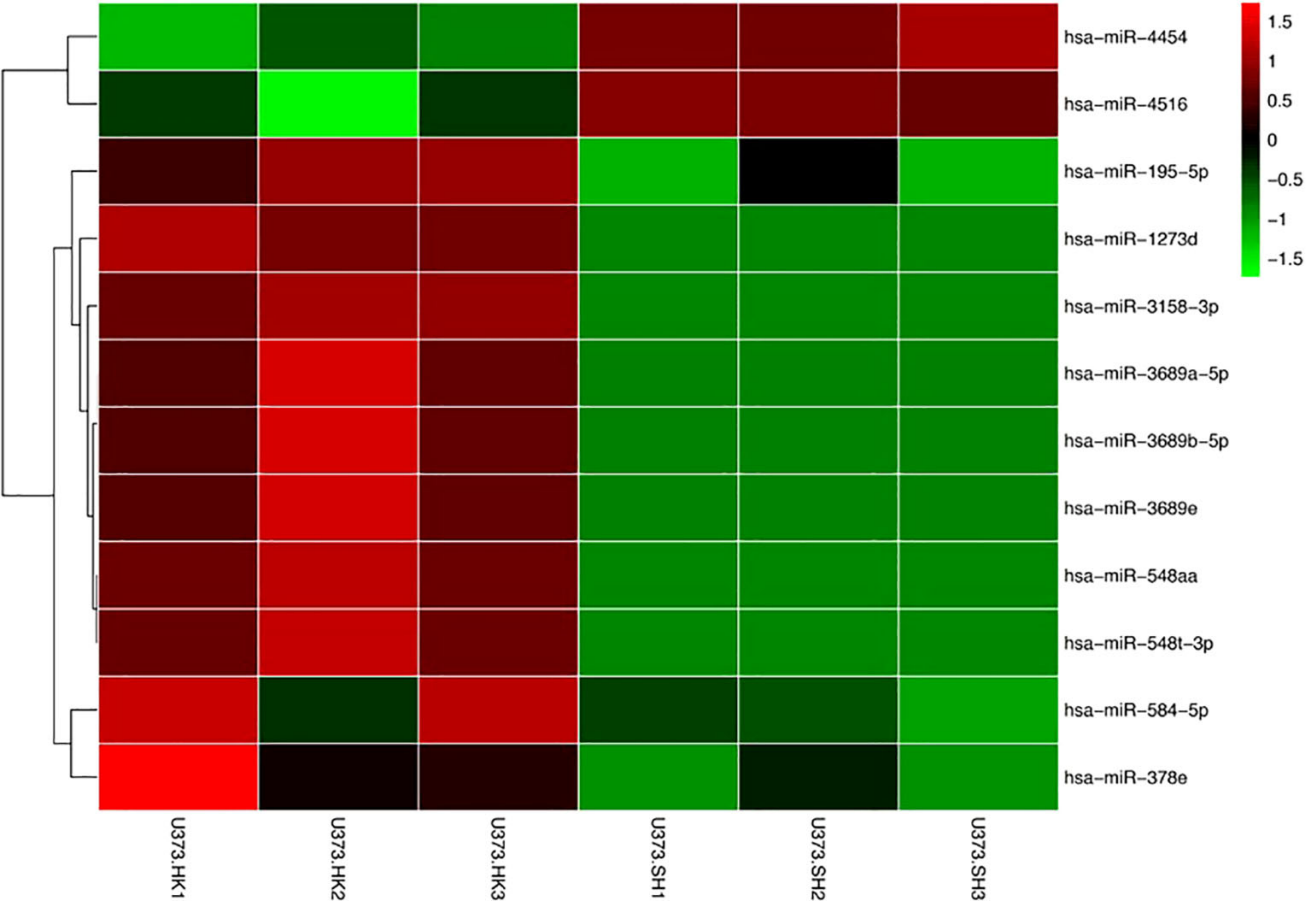
Identification of differential exosome miRNAs between U373-HK and U373-SH cells. Differential miRNAs were visualized, with the scale from the least abundant to the highest range being from −1.5 to 1.5. Their phylogenetic relationships are shown on the *left tree*.

We have previously published the verification of SPHK2 knockdown in our earlier article (11). The exosome sRNA was isolated and the gene expression profiles analyzed to identify the differentially expressed miRNAs between U373-HK and U373-SH cells. We found 12 exosome miRNAs differentially expressed between U373-HK and U373-SH cells. The heatmap showed that miRNA-4454 and miRNA-4516 were upregulated in glioma cells with SPHK2 knockdown, while miRNA-195-5p, miRNA-1273d, miRNA-3158-3p, miRNA-3689a-5p, miRNA-3689b-5p, miRNA-3689e, miRNA-548aa, miRNA-548t-3p, miRNA-548-5p, and miRNA-387e were downregulated in glioma cells with SPHK2 knockdown (**Figure 2**). We speculate that the expression and the transportation of these exosome miRNAs were affected by SPHK2 and that these miRNAs could mediate macrophage polarization through exosomes entering macrophages.

### GO classifications

**Figure 3:**
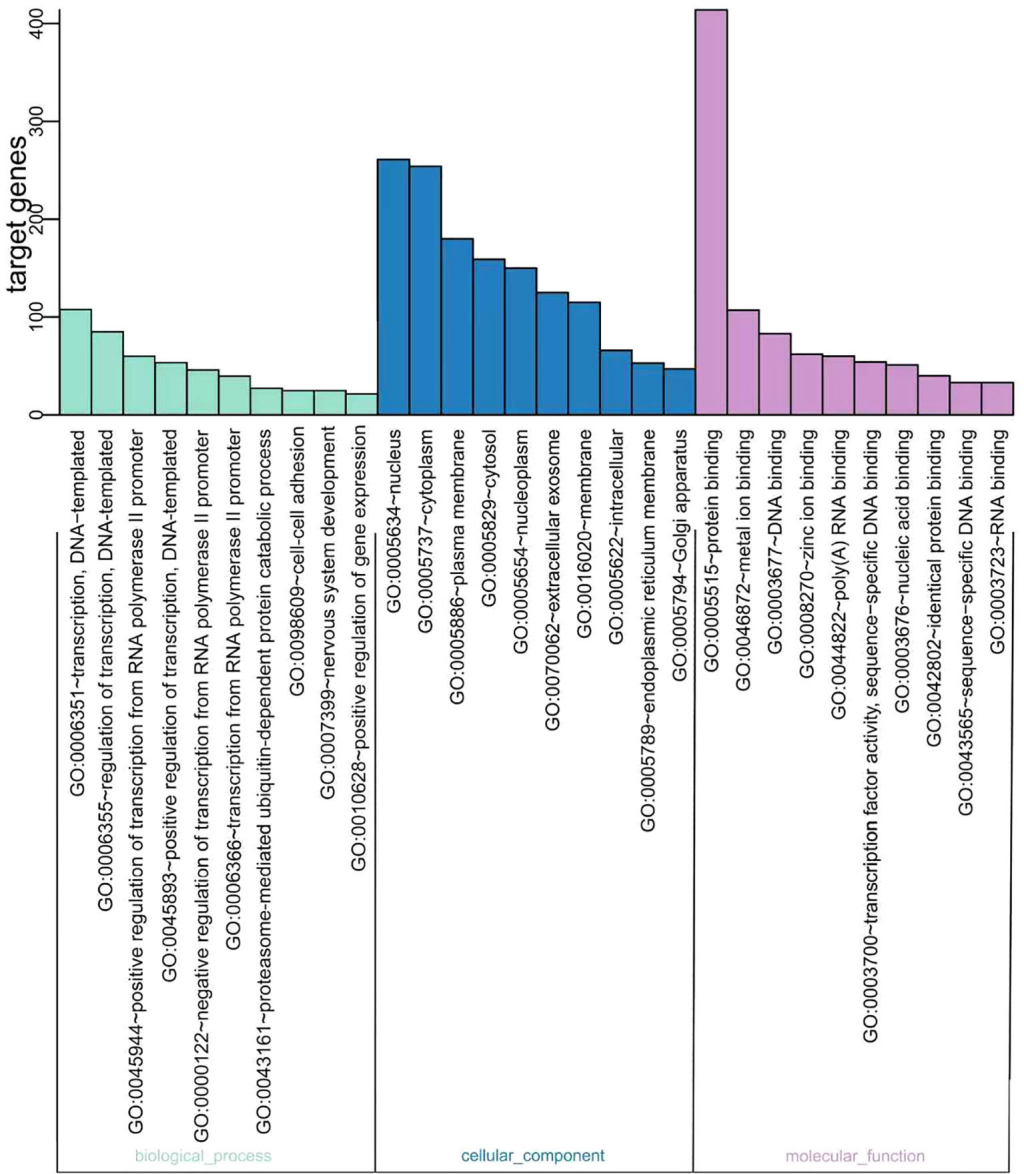
Gene Ontology (GO) functional classification of the miRNA targeting genes. The distributions are summarized into three main categories: biological process, molecular function (MF), and cellular component (CC). The *x*-axis indicates the different GO terms, while the *y*-axis indicates the number of genes in each category.

GO is a universally acknowledged gene functional enrichment database and is generally applied to determine enriched GO terms (17). Therefore, GO analysis was performed using hypergeometric distribution to obtain the most significant function of a series of genes (18, 19). A total of 12 miRNAs were assigned to 118 GO terms, including 53 biological processes, 30 cellular components, and 35 molecular function terms. Typical enriched GO terms are shown in **Figure 3**. The GO terms of the molecular function category were concentrated in “protein binding” (414 genes, 54.83%). The highest percentages of GO terms under the cellular component class were “nucleus” (261 genes, 34.57%) and “cytoplasm” (254 genes, 33.64%). The most prevalent “biological process” assignment was “transcription, DNA-templated,” including 106 genes and accounting for 14.04% of all genes. Moreover, other important assignments, such as “regulation of transcription, DNA-templated” (84 genes, 11.13%) and “positive regulation of transcription from RNA polymerase II promoter” (57 genes, 7.55%), were highly enriched, suggesting that glioma cells could alter the transcription of other cells around them through exosome miRNAs.

### The “Wnt signaling pathway” was significantly enriched by KEGG mapping

**Figure 4:**
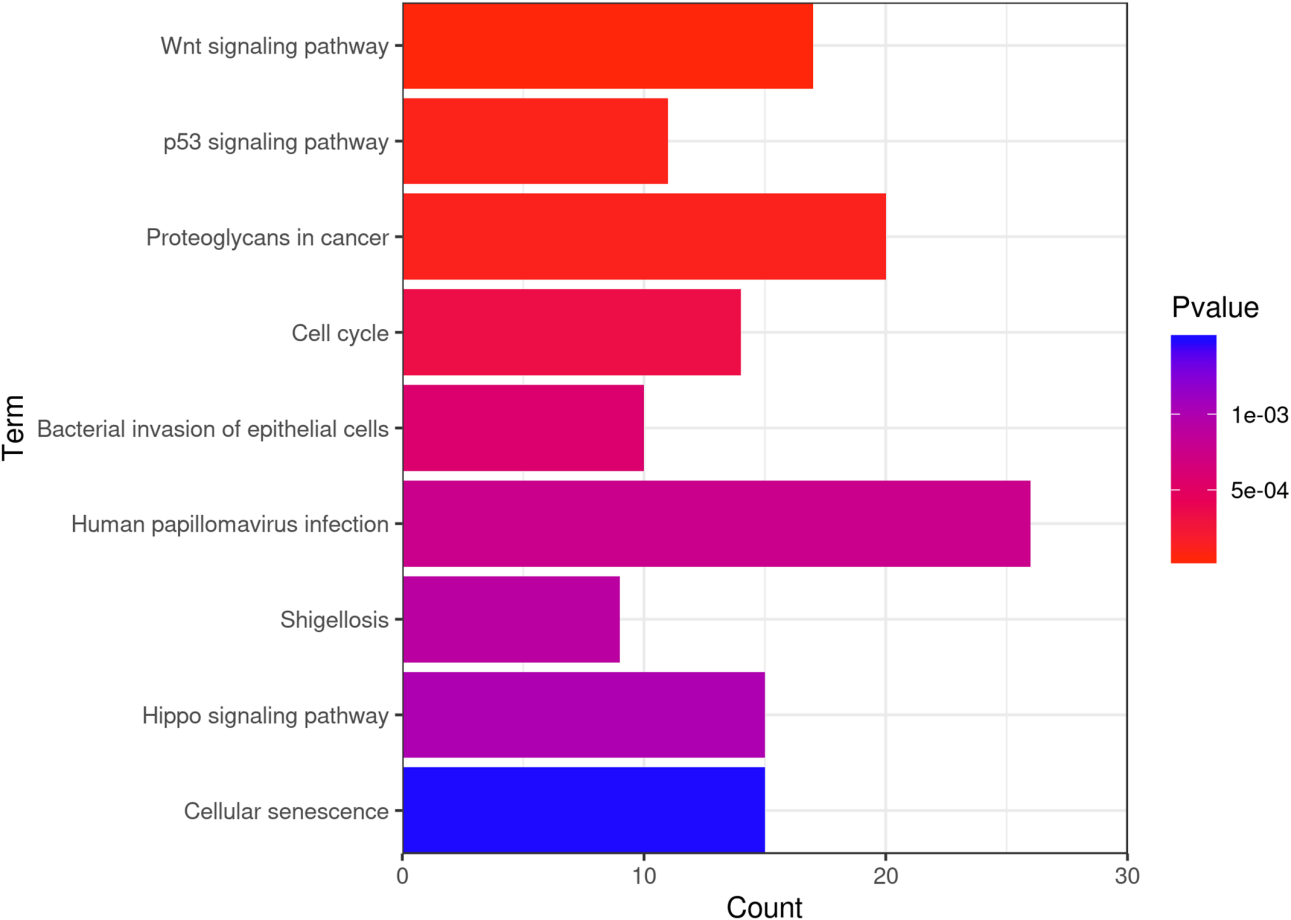
Bar chart of the results of the enriched Kyoto Encyclopedia of Genes and Genomes (KEGG) pathways. The *color* and the *size of the dots* represent the range of the *p*-values and the number of genes mapped to the indicated pathways, respectively. All 12 miRNAs targeting genes in the enriched pathways are shown.

**Figure 5:**
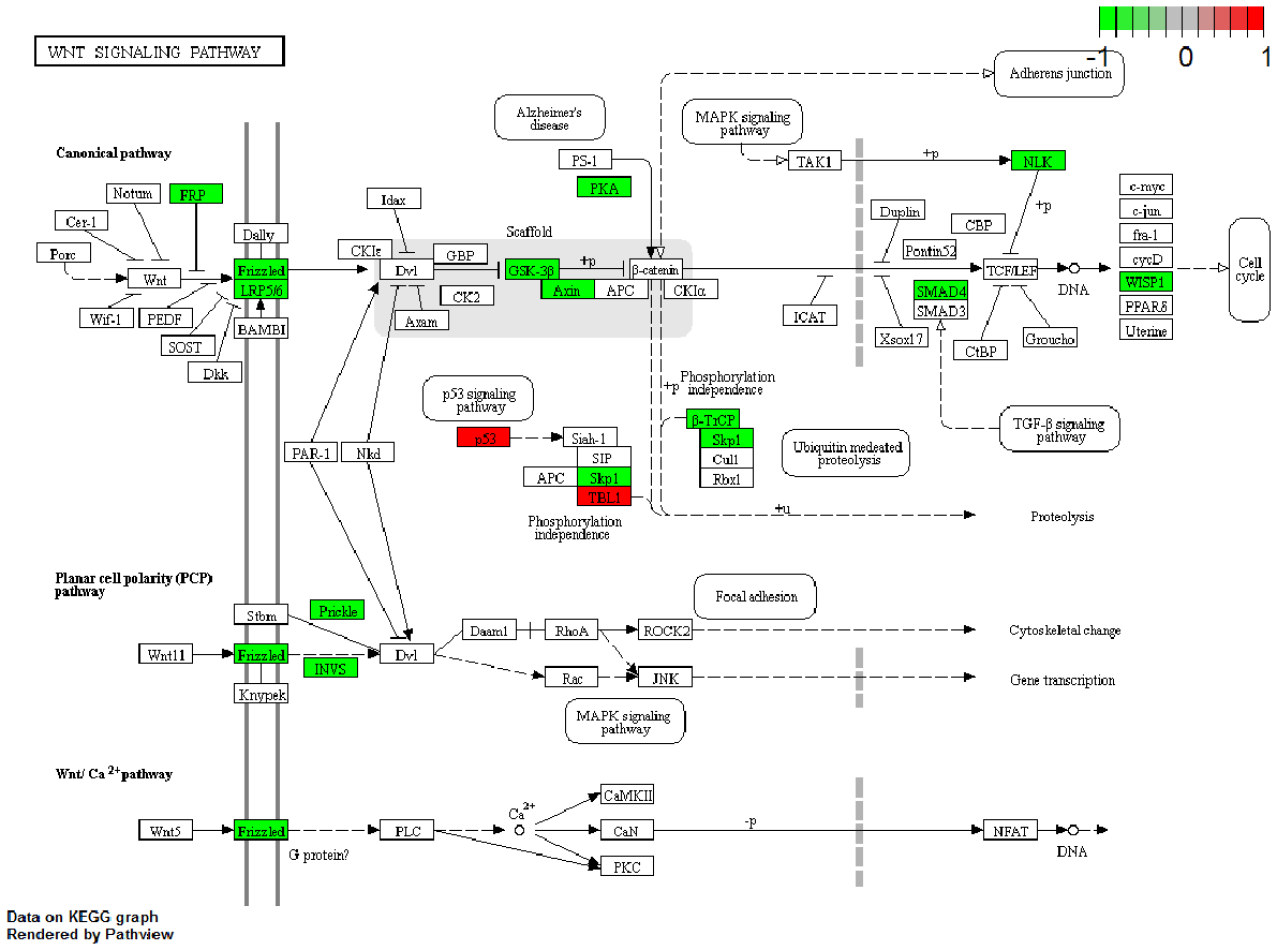
Gene expression profile of the “Wnt pathway.” The map of the “Wnt pathway” was modified from the Kyoto Encyclopedia of Genes and Genomes (KEGG) map. The *red boxes* indicate the upregulated genes, while the *green boxes* indicate the downregulated genes identified by KEGG mapping.

The KEGG database is a collection of various pathways representing the molecular interactions and reaction networks (20). To identify the downstream signaling pathways regulated by the identified exosome miRNAs, we mapped the KEGG database and found that the identified genes were significantly enriched in nine KEGG pathways, as shown in **Figure 4**. The genes were highly clustered in several signaling pathways, such as “Wnt signaling,” “p53 signaling,” “proteoglycans in cancer,” and “cell cycle,” suggesting that exosome miRNAs may perform their function through these pathways. “Wnt signaling” was identified as the most significantly enriched pathway in the KEGG analysis (gene ratio = 17/292, *p* < 0.001). A total of 17 genes were enriched in this pathway (**Figure 5**). Moreover, 15 genes (i.e., *SKP1*, *GSK3B*, *LRP6*, *BTRC*, *CCN4*, *INVS*, *SMAD4*, *SFRP1*, *NLK*, *FZD1*, *FZD2*, *FZD7*, *PRKACB*, *AXIN2*, and *PRICKLE2*) were upregulated and two genes (i.e., *TBL1XR1* and *TP53*) were downregulated (**Figure 5**).

## Discussion

Building on previous findings, this study aimed to determine whether SPHK2 regulates the release of exosomal miRNAs from glioma cells and to identify specific miRNAs that could potentially participate in macrophage polarization. The foundation for this inquiry includes reports that SPHK2 knockdown suppresses GB proliferation (21) and our own observation linking SPHK2 to TAM infiltration and glioma progression (10). While we have shown that miR-137 can drive M1 polarization by targeting SPHK2 (16), other studies have indicated a broader role, as SPHK2-deficient xenografts fail to polarize macrophages toward an anti-inflammatory phenotype and to exhibit a stunted growth (9). Therefore, the collective evidence positions SPHK2 as a key regulator of glioma growth through its influence on the polarization of TAMs.

Exosomes have emerged as key vehicles for intercellular communication, executing their pleiotropic functions through the targeted delivery of diverse cargo, including multiple RNA classes (both coding and non-coding), proteins, and lipids (22–24). In oncology, a critical manifestation of this process is the dynamic cross-talk between malignant cells and TAMs, which facilitates tumor progression and invasion while simultaneously offering novel avenues for therapeutic interventions (25). Supporting the functional relevance of exosomal cargo, the contents of Panc-1 cell-derived exosomes have been demonstrated to promote the polarization of J774.A1 macrophages toward an M1 phenotype (26). In light of these observations, we hypothesize that a key mechanism through which SPHK2 regulates TAM polarization is via the specific modulation of the exosomal miRNA cargo.

Our analysis revealed that SPHK2 knockdown altered the expression of 12 exosomal miRNAs in glioma cells. The 1,187 genes predicted to be targeted by these miRNAs were subjected to GO functional clustering. The results indicated that the major functional categories were “protein binding” (encompassing 414 genes, 54.83%), “nucleus” (261 genes, 34.57%), and “transcription, DNA-templated” (**Figure 3**). This GO-based functional profiling elucidates the potential mechanistic roles of the SPHK2-regulated miRNA cohort, thereby furnishing a foundational resource for further mechanistic studies.

Our KEGG mapping implicated the significant enrichment of multiple signaling pathways, including “Wnt signaling,” “p53 signaling,” “proteoglycans in cancer,” and “cell cycle” (**Figure 4**). Further analysis established the “Wnt signaling pathway” as the most significantly altered (**Figure 5**). Within this pathway, we observed the upregulation of 15 genes (i.e., *SKP1*, *GSK3B*, *LRP6*, *BTRC*, *CCN4*, *INVS*, *SMAD4*, *SFRP1*, *NLK*, *FZD1*, *FZD2*, *FZD7*, *PRKACB*, *AXIN2*, and *PRICKLE2*) and the downregulation of two genes (i.e., *TBL1XR1* and *TP53*), thereby pinpointing a specific set of candidate genes for future mechanistic studies.

The genes identified through KEGG analysis are compelling candidates that may play a role in macrophage polarization. For instance, *GSK3B* has been shown to phosphorylate and inactivate phosphatase and tensin homolog (PTEN), thereby potentiating the PI3K/Akt signaling pathway and promoting M2 polarization of the microglia (27). Similarly, regulatory T cells (Tregs) have been reported to alleviate inflammatory injury after intracerebral hemorrhage by shifting the microglia/macrophage polarization toward the M2 phenotype via the IL-10/*GSK3B*/PTEN axis (28). The expression of *WISP1* has also been linked to tumor purity and immune cell infiltration—particularly monocyte–macrophage trafficking and M2 polarization—although its role appears context-dependent. In some cancers, a higher *WISP1* expression correlates with better prognosis and a reduced M2 macrophage infiltration (29). Furthermore, studies on Sfrp1-deficient murine macrophages treated with recombinant *SFRP1* revealed a decreased expression of the M2 marker Egr2 and an increased expression of the M1 marker Gpr18 (30). Based on this literature and our bioinformatics findings, we propose a hypothetical model: SPHK2 may promote TAM polarization toward the M2 phenotype by modulating the exosomal miRNA cargo, which in turn could influence the expression of key regulators such as *GSK3B*, *WISP1*, or *SFRP1*. This model provides a testable framework for future research. To determine which of these genes functionally contributes to M2 polarization, real-time PCR and Western blot analyses will be performed in subsequent studies.

## Conclusion

In conclusion, our study establishes that SPHK2 knockdown in glioma cells significantly reshapes the exosomal miRNA cargo, underscoring its role as a pivotal regulator of miRNA sorting and secretion. We delineated a subset of miRNAs (e.g., miR-195-5p) whose export is enhanced by SPHK2, as well as others (e.g., miR-4454 and miR-4516) whose export is suppressed. While the functional implications of these alterations require experimental validation, our KEGG analysis suggests a potential connection to the Wnt signaling pathway. Therefore, this work primarily forges a mechanistic link between SPHK2 and exosomal miRNA sorting, providing a foundational framework for future research to directly investigate the functional impact of these SPHK2-modified exosomes on recipient cells, such as macrophages.

## Data Availability

The original contributions presented in the study are included in the article/supplementary material. Further inquiries can be directed to the corresponding authors.
